# NMR and UV Studies of 4-Thio-2′-deoxyuridine and Its Derivatives

**DOI:** 10.3390/molecules16075655

**Published:** 2011-07-01

**Authors:** Xiaohui Zhang, Yao-Zhong Xu

**Affiliations:** 1 College of Environment and Chemical Engineering, Dalian University, Dalian 116622, China; 2 Department of Chemistry, the Open University, Walton Hall, Milton Keynes, MK7 6AA, UK

**Keywords:** NMR, UV, 4-thiothymidine, DNA

## Abstract

5-Substituted-4-thio-2’-deoxyuridine nucleosides have been chemically synthesized and studied by NMR and UV spectroscopy. The results have been analyzed and discussed in connection with the previous data. The imino proton signal and the carbon signal of the thiocarbonyl group in the 5-substituted-4-thio-2’-deoxyuridines were found to be at much lower field, offering a potential for monitoring these modified bases at the DNA level. All 4-thionucleosides have strong absorptions at around 340 nm and consequently would be useful as potential UVA-induced anticancer agents.

## 1. Introduction

Cancer is one of major killers in modern society. The fundamental cause for cancer incidence is DNA damage and mutation [1]. An agent or treatment that can cause DNA damage can also be used as a therapeutic means by destroying cancerous cells. This, in fact, is the principle underlying chemotherapy and radiotherapy. However, often such treatments are either too toxic (in the case of chemotherapy) or too powerful (in the case of radiotherapy) and thus indiscriminate, also causing harm to normal cells. Clearly, improved treatments for cancer patients are urgently required. Recently we reported that 4-thiothymidine (thioT), an analogue of the naturally occurring nucleoside thymidine, can be incorporated into cellular DNA and activated by UVA light to kill cells [2,3]. These findings offered a new therapeutic approach to cancer treatment. This mild and synergistic approach has the advantage over conventional therapies, that it can target proliferating cells more selectively. As a part of our ongoing work, several 5-substituted-4-thio-2’-deoxyuridine derivatives (including 5-bromo-4-thio-2’-deoxyuridine [4] and 5-iodo-4-thio-2-deoxyuridine) were synthesized and their physico-chemical properties explored by NMR and UV spectroscopy. Interestingly, a great number of 5-substituted-2’-deoxyuridines have been synthesized and tested for *anti*-viral or *anti*-cancer activity [5,6]. However, to our surprise, only very few 5-substituted-4-thio-2’-deoxyuridines have been prepared [7,8,9,10,11] and little biological exploitation carried out [8]. Here we present our main findings on this topic and review their useful properties in relation with other thio-analogues.

## 2. Results and Discussion

**Scheme 1:**
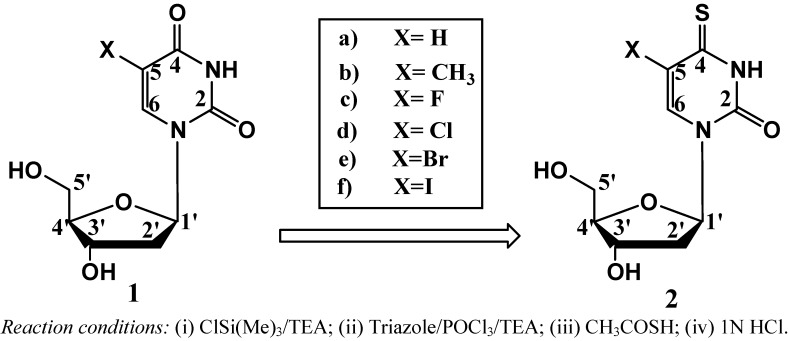
Chemical transformation of 5-substituted nucleosides to its 4-thioanalogues.

**Figure 1:**
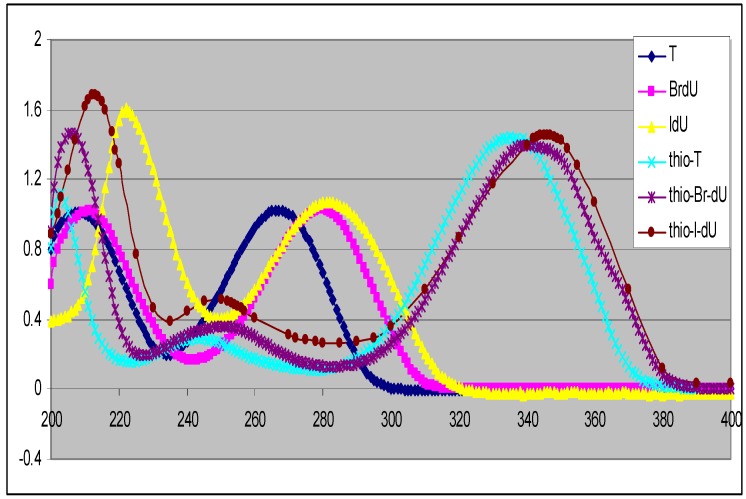
UV spectra of 5-substituted-2’-deoxyuridines (in CH_3_OH) and their 4-thioanalogues(inCH_3_CN).

Previously we reported a simple method for preparation of 4-thiothymidine by replacing the 4-oxygen of thymidine with sulfur [10]. More recently we extended the method to preparation of 5-bromo-4-thio-2’-deoxyuridine [4] and others. A general scheme for the preparation of 5-substituted-4-thio-2’-deoxyuridines is shown in Scheme 1. 2’-Deoxyuridine (**1a**) and its 5-substituted compounds (**1b–1f**) were converted to their 4-thio-analogues (**2a–2f**) by simply replacing oxygen atom at the 4-position with sulfur. These conversions are minimal modifications of 5-substituted-2’-deoxyuridine, thus possessing a good chance for the 4-thioanalogues to be incorporated into cellular DNA. Interestingly, the replacement of oxygen atom with sulfur shifts the UV spectra of the corresponding nucleosides to longer wavelengths with maxima at around 330–340 nm (the UVA range) as shown in Figure 1. The substituent at the 5-position also influences UV maxima: H (262 nm in H_2_O) [12], CH_3_ (267 nm in H_2_O) [12], F (265 nm in CH_3_CN) [13], Cl (277 nm in MeOH) [14], Br (279 nm in MeOH) [14] and I (285 nm in CH_3_OH) [14] for 5-substituted-2’-deoxyuridine. A red shift around 60–70 nm was found for 5-substituted-4-thiouridines and increased along with the conjugation capability of the substituents from H (332 nm in CH_3_CN), CH_3_ (336 nm in CH_3_CN), F (336.4 nm in H_2_O) [15], Cl (339 nm in H_2_O) [16], Br (340 nm in CH_3_CN) to I (345 nm in CH_3_CN). Clearly all these 4-thioanalogues (**2**) are sensitive to UVA light, offering therapeutic potential as UVA-induced drugs [2,3].

^1^H-NMR: Table 1 lists the ^1^H-NMR chemical shifts of the 5-substituted 2’-deoxyuridines and their 4-thioanalogues synthesized in our lab and some related compounds from the literature. Nucleosides consist of a sugar (deoxyribose) and a base. Since the modification is made on the base, chemical shifts of the base protons are substantially different, in particular, the 3-position imino group (NH). On the other hand, the chemical shifts of the sugar moieties are little changed.

**Table 1:** ^1^H-NMR of 5-Substituted-2’deoxyuridines and their 4-thioanalogues (in DMSO-*d*_6_).

	CH_3_	1’-H	2’-H	3’-H	4’-H	5’-H	3’-OH	5’-OH	6-H	NH
**1a**	5.67(5-H)	6.12	2.47	4.21	3.76	3.54	5.24	5.01	7.84	**11.28**
**1b**	1.79	6.15	2.49	4.22	3.74	3.55	5.21	5.01	7.68	**11.25**
**1c [13]**		6.12	2.10	4.25	3.78	3.62	5.25	5.15	8.21	**11.82**
**1d [14]**		6.11	2.13	4.25	3.80	3.61	5.25	5.16	8.32	**11.83**
**1e**		6.08	2.12	4.22	3.78	3.58	5.25	5.17	8.38	**11.78**
**1f**		6.08	2.10	4.22	3.78	3.58	5.23	5.14	8.38	**11.65**
**2a**	6.30(5-H)	6.07	2.11	4.22	3.81	3.56	5.26	5.03	7.78	**12.68**
**2b**	1.96	6.10	2.14	4.24	3.79	3.59	5.27	5.10	7.89	**12.69**
**2c [15]**		6.11	2.6-2.2	4.30	3.87	3.8-3.6	5.3-3.4	5.3-3.4	8.33	**12.80**
**2d [16]**		5.81	4.16	3.95	4.27	3.54	5.47	5.03	8.05	**13.10**
**2e**		6.02	2.21	4.22	4.09	3.61	5.26	5.21	8.52	**13.08**
**2f**		6.01	2.17	4.23	3.81	3.59	5.25	5.18	8.55	**12.99**

Data for 5-fluoro-2’-deoxyuridine (**1c**), 5-chloro-2’-deoxyuridine (**1d**), 5-fluoro-4-thio-2’-deoxyuridine (**2c**) and 5-chloro-4-thiouridine (**2d**) are from references [13,14,15,16], respectively.

The presence of a thiocarbonyl group in **2** is evident from the appearance of NH and thiocarbonyl carbon signals in the ^1^H and ^13^C-NMR spectra respectively. Exchangeable signals in the δ 12.68–13.10 ppm range in the low-field part of ^1^H-NMR spectra, attributable to the N–H protons, suffort the structures of molecules **2**. The appearance of only one carbon signal in the δ_C_ 185.30–190.70 ppm region (characteristic for a thiocarbonyl group) together with an intense absorption band at 332–344 nm in the UV spectra (Figure 1), confirm the presence of the thiocarbonyl moieties in compounds **2**.

It is interesting to note that the ^1^H chemical shifts of the imino proton (NH) in all the thionucleosides **2** are substantially higher (at around 13 ppm) than those of the parent nucleosides **1** that resonate at δ 11.2–11.83 ppm (Table 1). This difference offers a valuable NMR window to detect the imino proton of thionucleosides, as in general there are no signals from normal nucleosides appearing at such a low field. In addition these NH-proton signals are exchangeable and readily identifiable by D_2_O exchange experiments. Therefore these would also be a good marker in NMR studies of 4-thionucleosides and their corresponding DNAs.

^13^**C-NMR**: All the ^13^C signals were assigned using the standard procedure or DEPT protocols or 2-D NMR spectroscopy where necessary. A careful inspection of the ^13^C-NMR data (Table 2) provides useful structural information. As the modification occurs on the base, this is also reflected in the ^13^C chemical shifts of the base. There are four carbons on the base. The carbon at C-2- position (*i.e.*, 2-C) is substantially away from the modified positions (4 and 5), thus the chemical shifts of C-2 vary little. The most notable differences between the ^13^C-NMR spectra of **1** and **2** are the chemical shifts of the carbon at the 4-position (*i.e*., 4-C), and a characteristic downfield shift (from δ_C_ around 160 to δ_C_ around 190 ppm) was observed. This could be due to increased conjugation by the replacement of oxygen with sulfur at the 4-position. Interestingly, 2-thio-2’-deoxynucleosides differ only slightly while 4-thio-2’-deoxynucleosides exhibit somewhat large differences [8]. As shown in Figure 1, the introduction of a sulfur atom at the 4-position in the nucleosides shifts their UV spectra towards longer wavelength with maxima at 330 to 340 nm. In contrast, the UV spectra of 2-thio-2’-deoxyuridine analogues are similar to those of their un-modified nucleosides. For instance the UV λ_max _of 2-thio-2’-deoxyuridine is at273 nm (in H_2_O) [17] which is only slightly changed from that of its parent compound **1a** (UV λ_max_ at 262 nm). Due to the energy level of lone pair electrons of 3p in sulfur is higher than that of lone pair electrons of 2p in oxygen, the requied energy of the n→π^*^ transition for a thiocarbonyl is lower than that for a carbonyl. Although the spectra of 2-thionucleosides containing C=S bonds would be expected to shift to longer wavelengths relative to those containing C=O bonds, the thiocarbonyl in 2-position is attached to two nitrogens, so the UV absorption wavelength should also move back to shorter wavelengths. Overall, in the end there is a little change in the UV absorption maxima for the 2-thionucleoside analogues. This is also one of the reasons why 2-thionucleoside analogues are not exploited as useful agents for UVA-induced DNA damage.

**Table 2:** ^13^C-NMR of 5-Substituteduridine derivatives and their 4-thioanalogues (in DMSO-*d*_6_).

		CH3	1’-C	2’-C	3’-C	4’-C	5’-C		2-C	4-C	5-C	6-C
**1a**		--	84.11	39.63	70.43	87.40	61.29		150.45	163.14	101.76	140.53
**1b**		12.26	83.73	39.40	70.43	87.24	61.33		150.46	163.75	109.36	136.12
**1c [18]**		--	86.99	42.05	72.15	89.04	62.83		151.03	159.90	142.03	126.44
**1d [16]**		--	91.18	N/A	74.78	87.55	61.12		149.51	158.93	106.63	138.53
**1e**		--	84.90	40.18	70.01	87.61	60.85		149.82	159.28	95.76	140.37
**1f**		--	84.65	40.19.	70.01	87.52	60.82		150.12	160.51	69.27	145.05
**2a**	--		85.00	N/A	70.10	87.70	61.00	147.70		190.00	112.60	135.90
**2b**	17.00		84.70	39.90	70.10	87.70	61.00	147.80		190.70	117.70	133.50
**2c [23]**	--		87.66	80.22	72.97	69.82	62.76	146.39		179.86	147.23	118.16
**2d [16]**	--		92.27	79.51	75.04	88.78	61.18	147.03		185.30	116.37	135.45
**2e**	--		85.63	39.23	69.43	87.78	60.40	147.19		186.39	106.63	137.29
**2f**	--		85.39	39.50	69.50	87.74	60.41	147.63		189.31	82.99	140.15

Data for 5-Fluoro-2’-deoxyuridine (**1c**), 5-Chloro-2’-deoxyuridine (**1d**), 5-Fluoro-4-thiouridine (**2c**) and 5-Chloro-4-thiouridine (**2d**) are from references [16,18] respectively).

As the major factor in ^13^C chemical shift is paramagnetic shielding, that is different from diamagnetic shielding in ^1^H chemical shift, thus the shielding constant σ for ^13^C spectrum is generally expressed as below [19]:

σ_p_ = *−e^2^h^2^ (ΔE)^−1^*{*r^−3^*}_2p_[*Q*_AA_ + Σ_B_*Q*_AB_] / *2m^2^C^2^*

or σ_p _∝ *−(ΔE)^−1^*{*r ^−3^*}_2p_

where C is the light speed; m and e are respectively for the mass of atom and number of electron; the term {r^−3^}_2p_ is the mean inverse cube of the distance from the nucleus for the carbon 2p atomic orbit; This term accounts for the charge density effect in the paramagnetic term since increasing charge density leads to an expansion of the orbital and thereby a reduction in σ_p_; ΔE represents the excitation energy of average electron to the low lying excited state; Σ_B_ goes over all atoms; Q is bond order; Q_AA_ is contribution of electron density of 2p orbital in nucleus; Q_AB_ is bond order between nucleus and bond which relate to nucleus.

Comparing the carbonyl oxygen group in a nucleoside with the thiocarbonyl sulfur group in a thionucleoside would lead to the prediction that σ_p_ should be more for the latter if the {r^−3^}_2p_ term dominates since there is a less contribution from electronegativity on sulfur, the deshielding of carbon atom is strong and the signal carbon resonate moves to lower field such as the ^13^C-NMR spectra of δ_C_ = 159.28–163.75 ppm for C=O in compound **1** and δ_C_ = 179.86–190.70 ppm for C=S in compound **2**.

Obviously, the equation shows that the ׀σ_p_׀ dependence is mainly affected by the excitation energy of an average electron ΔE to paramagnetic shielding under consideration; that is, the bigger ׀σ_p_׀, the stronger paramagnetic shielding, and the atom will resonate at lower field. Therefore, σ_p_ depends uponΔE. In addition, it has been shown by Figgis *et al.* [20] that organic carbonyl oxygen shieldings correlate approximately with λ_max_^n→π*^ values (*i.e.*, the inverse of the excitation energy for the lowest electronic transition). For C=O, it is n→π^*^ absorption, the absorption of UV is 280 nm, (ΔE)^−1^ which is bigger makes ׀σ_p_׀ bigger, the deshielding of the carbon atom is stronger, and the signal carbon resonance moves to lower field. For C=S, it is also n→π^*^, the absorption of UV moves towards longer wavelength, approaching to 400 nm, the intensity of absorption in C=S is bigger than that in C=O, so the wave of absorption is longer. For ^13^C-NMR, the C=S resonance is downfield in comparison with that in a C=O.

Since the C=C double bond carries a polar group, the electron distribution is thus displaced. This displacement is usually understood as a combination of inductive effects and conjugative effects. There is the electron excited state as n-π* or π-π* in molecules where C=C double bond attached a polar group when highly electronegative atoms like halogen are near these sigma σ electrons. The highly electronegative atoms attract these sigma σ electrons toward themselves and away from the spinning nuclei. These would affect on the deshielding and an NMR signal to be generated would move further downfield.

**Figure 2:**
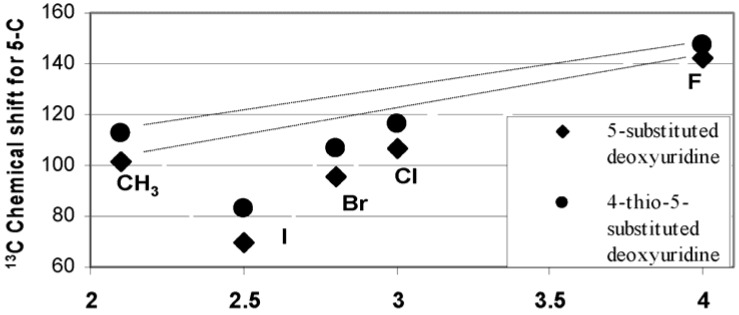
^13^C chemical shift of the atom (group) at 5-C plotted against its electro negativity.

Table 2 shows that the 5-C signals of the fluorine-modified deoxyuridine **1c** and its thioanalogue **2c** are substantially different from other halogen-modified compounds **1d**, **1e**, **1f** and their 4-thioanalogues **2d**, **2e**, **2f** respectively. Figure 2 plots ^13^C chemical shifts of C-5 against with the electronegativity of the 5-substitutents. The greater the electronegativity of atom or group, the lower the electron density around C–X and the further downfield the chemical shift. When linked with fluorine atom, the 5-C has the highest values, namely 142.03 ppm (for **1c**) and 147.23 ppm (for **2c**). This could be ascribed to the extremely high electronegativity of fluorine. F, Cl, Br and I are each more electronegative than the H atom, it could be anticipated that, when the H atom of C-5 are replaced by these substituents, the ^13^C resonance would be progressively shiftded to much lower field. Obviously, the electron-withdrawing inductive effects of the 5-substituent do play an important role for F, Cl and methyl group. However when a substituent at the 5-position is I or Br, the chemical shift of the 5-C is even lower than that of those 5-C bearing H- or CH_3_ group. The ^13^C resonance of C-5 for BrdU (5-bromo-2’-deoxyuridine) and IdU (5-iodo-2’-deoxyuridine) is displaced to higher field relative to that of dU (2’-doxyuridine) by 6 ppm for BrdU and by 32.49 ppm for IdU. Clearly the electron-withdrawing effect alone is not enough to explain those. This unusual effect could be explained by “heavy atom effect” which invariably cause shifts upfield [21]. When a carbon atom is attached by heavy halogen atom (such as Br or I), the diamagnetic interactions arising from more electron charge within Br or I atoms may exhibit an abnormally high ^13^C shielding and cause the chemical shift of ^13^C resonance to upfield.

On the other hand, the circulating π electrons are in the delocalized pyrimidine ring. These π electrons create a magnetic field that is parallel to the lines of force of the external magnetic field. This will aid the external field (a deshielding effect) and allow the NMR signal generation to occur at a lower magnetic field strength setting. For example, the carbons at the 6-position of **1c** and **2c** have reduced values (δ_C_ 126.44 ppm for **1c** and δ_C_ 118.16 ppm for **2c**). These results can be explained by the conjugative effects which operate in the π-system and have π-donor group for p orbital in Cl, Br, I, causing an upfield shift. Therefore, as the conjugation to p orbital of Cl and Br elements usually has a smaller effect on C=C than that to p orbital of I element, so the signal of C-6 of iodo-substituted nucleosides (**1f** and **2f**) have high values than other halogen-substituted analogues.

Such a shift suggests possible changes of base-pairing properties compared with their parent nucleosides. The cause of this lower field shift may also reflect the longer conjugation of the ring system of thio-nucleosides. This is further evidenced from their longer wavelength absorption in their UV spectra (Figure 1). In general, deoxyuridine nucleosides have absorption maxima at 260–285 nm arising from their π→π^*^ transitions. Although several deoxyuridines containing an X group are found to have increased UV maxima and 4-thioanalogues have further increased UV maxima, there is still a need for new compounds absorbing at even long-wavelengths. We developed a method by replacing the oxygen atom by sulfur to produce UVA-sensitive thio-analogues as shown in Scheme 1 (above).

The replacement of an oxygen atom at the 4-position by selenium in uridine derivatives produces UVA-sensitive 4-seleno-uridine and its analogues [22]. The UV absorption spectrum for 4-seleno-2’-deoxyuridine was compared to that of 2’-deoxyuridine and 4-thio-2’-deoxyuridine. As expected, its absorption maximum was found at longer wavelength [368 nm (in H2O)] than those of 2’-deoxyuridine and 4-thio-2’-deoxyuridine. This is due to the fact the energy level of the lone pair on selenium is higher than those on a sulfur atom or an oxygen atom, (ΔE) which is smaller, leads to longer wavelength absorption. Therefore the 4-selenonucleosides would be more sensitive toward UVA light than 4-oxy- and 4-thio-nucleosides. This UVA absorption property should provide a potential to specially target molecules of seleno-modified DNA with UVA light at 368 nm.

Recently, much effort was directed to study the photophysical and photochemical properties of uridine derivatives that contain both C=O and C=S fragments. In the series of 4-thio-2’-deoxyuridines, it was found that the presence of the C=S function in the molecules dominated their photochemistry in aqueous solution [2,8,23,24]. According to the idea, 4-selenouridine derivatives that contain X groups (where X = halogen) will shift to much longer UV wavelengths than that of thiocarbonyl groups and could be particularly useful as potential UVA-induced anticancer agents.

In summary, the thiation of nucleosides and related compounds has been extensively studied in the past, the UVA-sensitive 4-thio-2’deoxyuridine and its derivatives have never been studied in detail by NMR and UV spectroscopy. We have now established the identities of the thio products derived from carbonyl products in NMR and UV spectroscopy.

## 3. Experimental

### 3.1. General

^1^H-Nuclear magnetic resonance (NMR) spectra and ^13^C-NMR spectra were recorded using a JEOL LA300 spectrometer at 300 and 75 MHz, respectively. ^1^H-NMR and ^13^C-NMR spectra were determined in DMSO-d_6_ solution and chemical shifts are quoted in parts per million (p.p.m.) from tetramethylsilane as internal standard. Ultraviolet spectra were recorded with a Philips PU 8700 UV/Vis spectrophotometer for 5-substituted 4-thio-2’-deoxyuridine derivative (50 mM in CH_3_CN).

### 3.2. Typical Thiation Chemical Preparation of 5-Substituted-4-thiouridine Derivatives

A general procedure is described for the chemical synthesis of 4-thio-2’-deoxyuridine (**2a**), 4-thiothymidine (**2b**), 5-bromo-4-thio-2’-deoxyuridine (**2e**) and 5-iodo-4-thio-2’-deoxyridine (**2f**). The appropriate 5-substituted-deoxyuridine derivative (16 mmol, ca. 3.9~5.7 g) was dissolved in dry THF (150 mL). Triethylamine (63.4 mL, 0.454 mol) was added, followed by trimethylchlorosilane (6.34 mL, 81.2 mmol). The reaction mixture was stirred in an ice bath for 2.5 h after all the materials were added. TLC showed no residual starting material at this time. The reaction mixture was diluted with 50% ethyl acetate in hexane (250 mL), washed with water (2 × 100 mL) and saturated aqueous NaCl (80 mL). The aqueous phase was back extracted with 50% ethyl acetate in hexane (1 × 150 mL). The combined organic phase was dried (Na_2_SO_4_), and evaporated to give crude product which was used directly in the next step.

1,2,4-Triazole (6.8 g, 98.4 mmol) was suspended in anhydrous CH_3_CN (80 mL) at 0 °C. POCl_3_ (2.1 mL), then triethylamine (16 mL) were added slowly. After 1 h, the 3’,5’-bis-(trimethylsilyl)-5-substituted-2’-deoxyuridine derivative (6.7 mmol) in CH_3_CN (30 mL) was added over 30 min. Then the solution was stirred for 16 h at room temperature and the reaction was monitored by TLC (solvent: 50:50 *n*-pentane/diethyl ether). After the starting material was converted into a new compound with lower R_f_, the reaction mixture was filtered, diluted with ethyl acetate (160 mL) and washed with saturated aqueous NaHCO_3_ (150 mL), then twice with 150 mL of saturated aqueous NaCl. The organic layer was dried over anhydrous Na_2_SO_4_ and the solvent evaporated. The residue was dried by repeated evaporation of a toluene solution to give product, then used in the next step.

To a solution of 3’,5’-*O*-(trimethylsilyl)-5-substituted-2’-deoxy-4-triazolyuridine derivative (6.0 mmol) in CH_3_CN (120 mL) was added thioacetic acid (6 mL, 6.39 g, 83.9 mol) in an ice bath. The reaction mixture was left stirring overnight and monitored with TLC (5% CH_3_OH in CHCl_3_ as eluent) until the starting material was completely converted into a new spot. The reaction solution was diluted with CH_2_Cl_2_ (250 mL), washed with saturated NaHCO_3_ (2 × 250 mL), then with saturated aqueous NaCl (250 mL). The organic layer was dried (Na_2_SO_4_) and evaporated under reduced pressure to give crude product, which was taken up in THF and acidified with 1 N HCl (pH = 3). The mixture was stirred at room temperature for 20 h and the reaction monitored by TLC. The desilylated 4-thio-2’-deoxyuridine product was purified by column chromatography on silica gel (eluting with 5% CH_3_OH in CHCl_3_) to afford the corresponding products **2a**, **2b**, **2e** or **2f**(1.39 g, 38%;, 1.69 g, 36%; 1.62 g, 31; 1.59 g, 27% overall yield from 5-substituted-2’-deoxyuridine derivatives, respectively).

## 4. Conclusions

5-Substituted-4-thio-2’-deoxyuridines can be effectively prepared from its parent nucleosides and have distinctive NMR and UV properties that can be used for easy monitoring and exploited as potential UVA-induced anticancer agents.
